# 14-3-3: A Case Study in PPI Modulation

**DOI:** 10.3390/molecules23061386

**Published:** 2018-06-08

**Authors:** Alice Ballone, Federica Centorrino, Christian Ottmann

**Affiliations:** 1 Laboratory of Chemical Biology, Department of Biomedical Engineering and Institute for Complex Molecular Systems, Eindhoven University of Technology, Den Dolech 2, 5612 AZ Eindhoven, The Netherlands; A.Ballone@tue.nl (A.B.); F.Centorrino@tue.nl (F.C.); 2 Department of Chemistry, University of Duisburg-Essen, Universitätsstrasse 7, 45117 Essen, Germany

**Keywords:** 14-3-3 PPIs, PPI modulation, small molecules

## Abstract

In recent years, targeting the complex network of protein–protein interactions (PPIs) has been identified as a promising drug-discovery approach to develop new therapeutic strategies. 14-3-3 is a family of eukaryotic conserved regulatory proteins which are of high interest as potential targets for pharmacological intervention in human diseases, such as cancer and neurodegenerative and metabolic disorders. This viewpoint is built on the “hub” nature of the 14-3-3 proteins, binding to several hundred identified partners, consequently implicating them in a multitude of different cellular mechanisms. In this review, we provide an overview of the structural and biological features of 14-3-3 and the modulation of 14-3-3 PPIs for discovering small molecular inhibitors and stabilizers of 14-3-3 PPIs.

## 1. Introduction

14-3-3 is a family of conserved eukaryotic regulatory proteins involved in a multitude of biological processes, such as signal transduction, viral and bacterial pathogenesis, apoptotic cell death, and cell cycle control. It was discovered in 1967 by Moore and Perez in brain tissue [1]; the name is derived from a combination of its fraction number (14) on DEAE-cellulose chromatography and its migration position in subsequent gel electrophoresis (3-3). There are seven mammalian 14-3-3 isoforms, named with Greek letters σ, ζ, β, γ, η, ε, and τ [2,3]. All isoforms share a high degree of sequence conservation among species [4]; however, the biological role in, for example, pathogenesis for each of the isoforms has not been completely understood [5,6].

**Figure 1:**
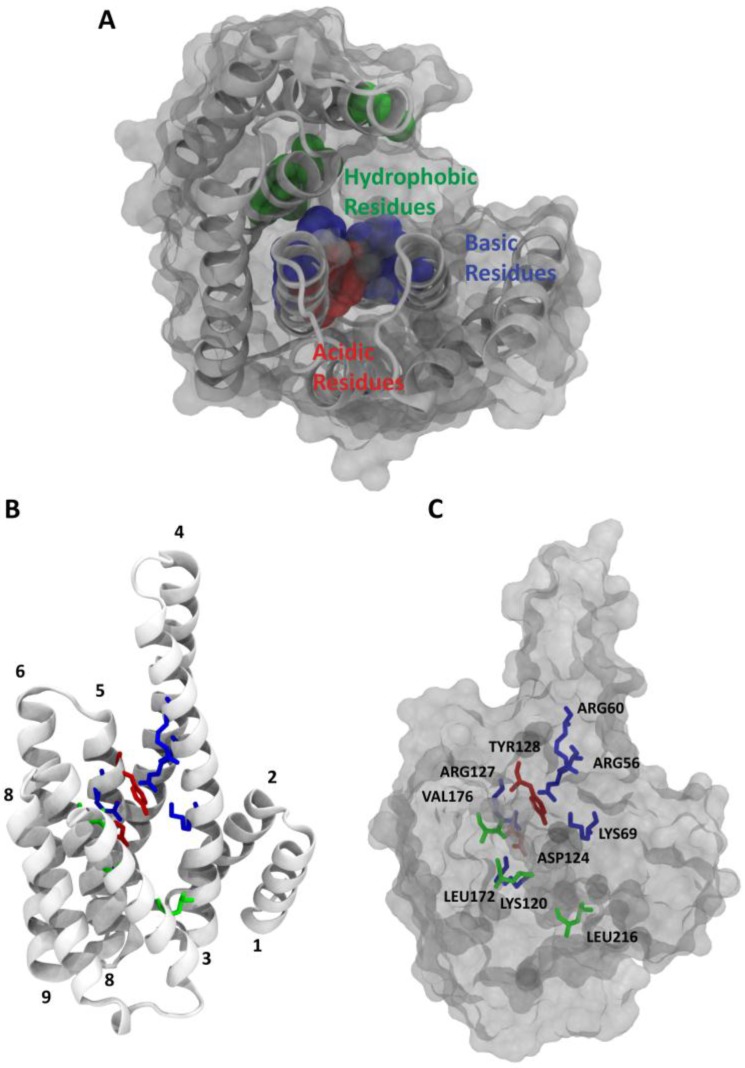
View of the monomer of the 14-3-3ζ isoform (PDB ID: 5EXA) [11] (**A**). The molecular assembly of the structural elements is shown in cartoon and surface representation. Hydrophobic, basic and acidic residues are depicted in green, blue, and red, respectively (**B**,**C**). View of the monomer turned by 90 degrees around the y-axis; helices from 1 to 9 constituting the classical 14-3-3 monomer are represented, as well as residues that belong to the phospho-acceptor side and residues that establish the hydrophobic interactions.

The 14-3-3 proteins exist mainly as dimers with a monomeric molecular mass of approximately 30,000 Da. Each monomer consists of a bundle of nine alpha helices organized in an antiparallel fashion. Its functional form is represented by the homo- or the heterodimer showing the typical W-like shape, which is formed by monomers of different isoforms [7,8]. Salt bridges and hydrophobic interactions are responsible for the stability of the dimeric structure [9]. The concave surface is characterized by an amphipathic groove in each monomer; on one side of the groove, helices 3 and 5 present a cluster of charged polar residues. On the other side of the groove, helices 7 and 9 present a patch of hydrophobic residues (Figure 1). The residues belonging to the groove are mostly conserved among the different isoforms of the 14-3-3 family, while there is more variation in the exterior of the W-shaped protein structure [10].

## 2. 14-3-3/Ligand Interactions

**Figure 2:**
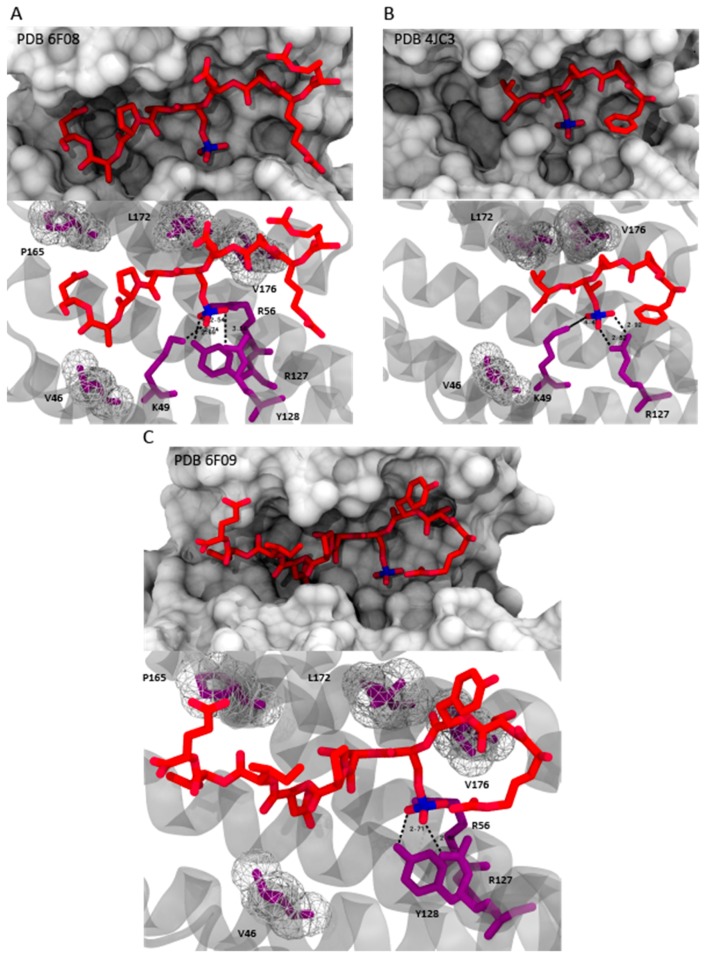
Common recognition motif for 14-3-3 proteins that contain a phosphorylated serine or threonine; mode II for the complex Son of sevenless homolog 1 SOS1/14-3-3ζ (PDB ID: 6F08) [20] (**A**). Higher side: 14-3-3ζ (white surface) and the peptide SOS1 (red sticks); lower side: Polar contacts (black dashed lines) and hydrophobic interactions (wireframe white spheres) between the residues of 14-3-3ζ (red sticks and white-transparent cartoon) and the pSer binding site of SOS1 (violet sticks). Mode III for the complex ERα/14-3-3σ (PDB ID: 4JC3) [21] (**B**). Higher side: 14-3-3σ (white surface) and the peptide ERα (red sticks); lower side: Polar contacts (black dashed lines) and hydrophobic interactions (wireframe white spheres) between the residues of 14-3-3σ (red sticks and white-transparent cartoon) and the pSer binding site of ERα (violet sticks). Mode I for the complex ubiquitin specific protease 8 (USP8)/14-3-3ζ (PDB ID: 6F09) [22] (**C**). Higher side: 14-3-3ζ (white surface) and the peptide USP8 (red sticks); lower side: Polar contacts (black dashed lines) and hydrophobic interactions (wireframe white spheres) between the residues of 14-3-3ζ (red sticks and white-transparent cartoon) and the pSer binding site of USP8 (violet sticks).

The 14-3-3 proteins have the ability to bind to many functional signaling protein (ligands), thereby mediating their physiological effect. In this context, the association/dissociation of these protein complexes becomes the key dynamic process for the regulation of different protein mechanisms. Depending on its interaction with specific protein partners, 14-3-3 participates in several regulatory processes, such as cell cycle control, survival signaling, cell adhesion and neuronal plasticity [12]. For this reason, growing the knowledge in the field of 14-3-3/ligand interactions can be defined as an important starting point for the development of new therapeutic approaches in drug discovery. The variability in the outside surface of the protein might define the specificity of the 14-3-3 proteins for their interaction ligand. Likely, the complex formation depends on binding of the consensus sequence to the amphipathic groove and concurrent recognition of outside amino acids by a partner protein. Of particular importance is the common determinant binding of a phosphorylated residue of the ligand that mediates the contact with 14-3-3. Two major consensus binding motifs are represented by the mode-I RSX-pS/T-XP peptide and the mode-II RXXX-pS/T-XP, where X is a generic amino acid (cysteine excluded), with pS/T standing for phosphorylated serine or threonine [13] (Figure 2). In particular, the side chains of Arg56, Arg129, Tyr130, and Lys49 of the conserved amphipathic groove form the primary phospho-accepting pocket within the binding channel [14,15]. Mode III motifs were later defined as C-terminal sequences, where the phosphorylated serine or threonine residue is the penultimate residue of the binding partner [16] (Figure 2). Currently, in several bioinformatics and biochemical studies, almost 600 interaction partners of 14-3-3 have been identified [17]. More than 40 14-3-3/ligand complexes have been solved by means of X-ray crystallography, which is thus a valuable tool for exploring structural features of this protein family [18,19].

## 3. 14-3-3 PPIs in Human Diseases

Protein–protein interactions (PPIs) are fundamental for a broad range of biological processes; the alteration in PPI events is among the main causes of diseases, such as cancer, diabetes, and others [23,24]. Most proteins do not function as single entities but are engaged in dynamic interactions with other proteins, constituting highly organized and responsive networks [25]. PPIs can result from the interaction between identical or non-identical chains (homo- or hetero-oligomers) and they can involve the same surface of both monomer (isologous assemblies) or different surfaces (heterologous assemblies) [26]. PPIs can also be distinguished as permanent or transient interactions. Permanent interactions are very stable and hence exist only in the complexed form. Transient interactions are temporary and typically require a set of conditions that promote the interaction, such as phosphorylation, conformational changes, or localization to specific areas of the cell. Transiently interacting proteins are involved in a wide range of cellular processes, and the specific modulation of these PPIs with small molecules has become a significant focus in drug discovery. With hundreds of known protein–protein interactions, the family of 14-3-3 proteins represents an especially interesting case for the study of PPI modulation. Moreover, 14-3-3 has not been directly linked to a specific disease, it has been implicated in a variety of biological processes, including cell cycle regulation, signal transduction, protein trafficking, and apoptosis [27], as well as cancer development and progression. Particularly, 14-3-3 proteins participate in phosphorylation-dependent protein–protein interactions that control progression through the cell cycle, initiation and maintenance of DNA damage checkpoints, activation of MAP kinases, prevention of apoptosis, and coordination of integrin signaling and cytoskeletal dynamics [28]. Besides neoplastic alterations, 14-3-3 proteins have been related to the development of neurodegenerative disease [29,30]. In this context, 14-3-3 was found to bind Tau [31,32,33], stimulating its aggregation in neurofibrillary tangles, which are abundant in patients affected by Alzheimer disease [34]. Recently, the role of 14-3-3 in other pathologies has emerged; for example, cystic fibrosis, through the regulation of the trafficking of the cystic fibrosis transmembrane conductance regulator (CFTR) protein [35]. The PPI interaction between 14-3-3 and the ubiquitin specific protease 8 (USP8) has recently been shown to exert a significant role in the pathogenesis of Cushing’s disease through the regulation of USP8 enzymatic activity [22,36]. Another relevant example is the GTPase activity of Ras, which is modulated through its protein–protein interaction with SOS1. Modulation of this interaction affects downstream PPI-mediated kinase activities within the entire pathway due to binding of 14-3-3 to SOS1 [20]. One more emerging area of investigation is presented by the role of 14-3-3 in parasite proliferation and survival and as potential vaccine candidates [37].

## 4. Stabilizers of 14-3-3 PPIs

**Figure 3:**
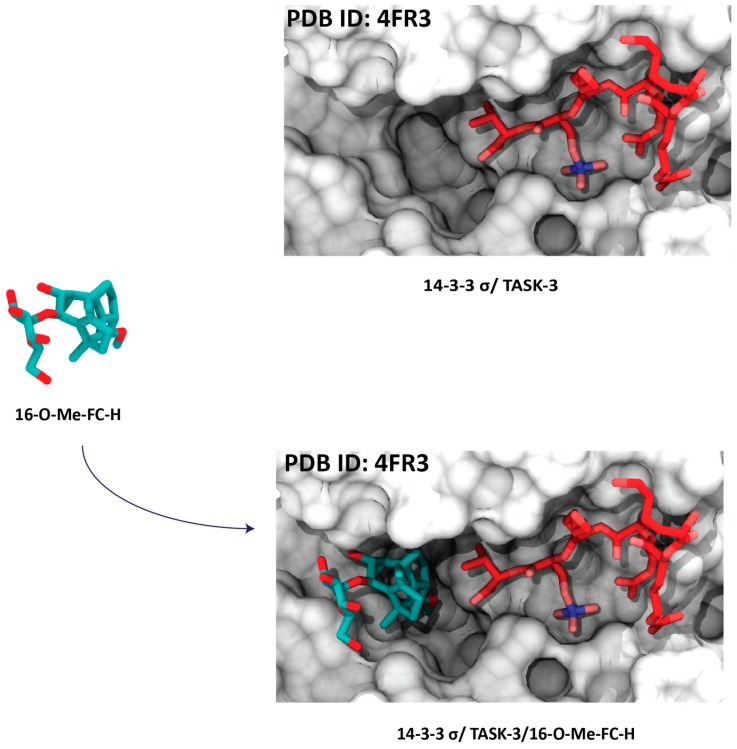
Example of a stabilizer that binds to the interface of 14-3-3 (white surface) and a peptide motif (red sticks) binding within the groove. When this peptide motif binds within the 14-3-3 groove, a druggable pocket is formed. A semisynthetic fusicoccin derivative (16-O-Me-FC-H) (cyan sticks) binds to this pocket and stabilizes the potassium channel subfamily K member 9 (TASK-3) and 14-3-3 motif complex. The information was obtained analyzing the PDB entry 4FR3 [39].

Protein–protein interactions (PPIs) are involved in all diseases and, nowadays, they are considered a significant topic in drug discovery. The modulation of these PPIs with small molecules represent a key strategy for designing novel bioactive compounds to enlarge the druggable genome (see example Figure 3). A number of very interesting natural and synthetic molecules have been reported in the literature that achieve their physiological activities by stabilizing either homo- or hetero-oligomeric complexes of their target proteins [38].

### 4.1. Natural Products

#### 4.1.1. Fusicoccin-A

**Figure 4:**
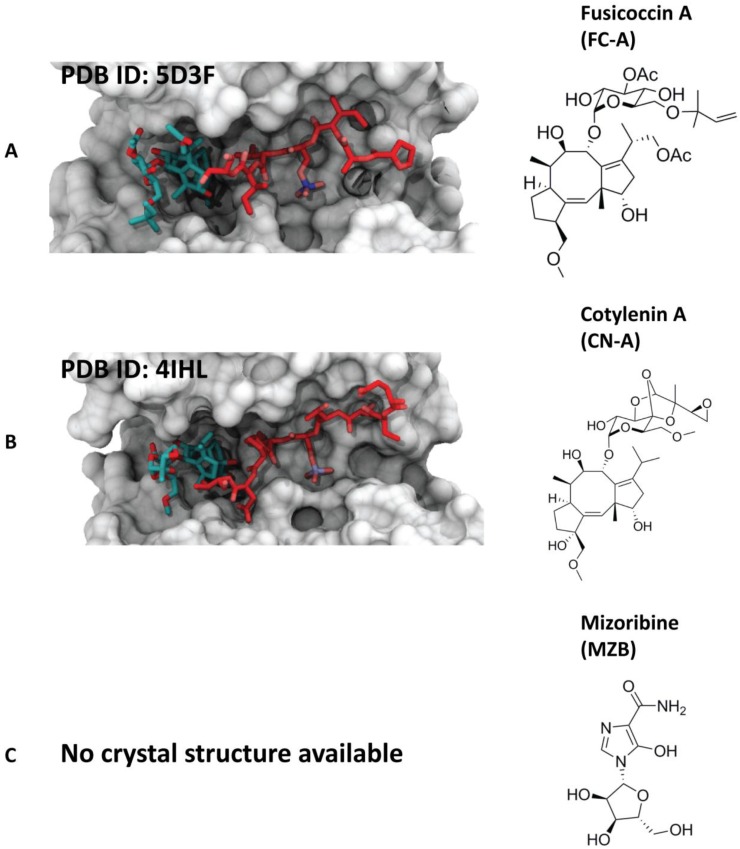
Examples of the different natural 14-3-3 protein–protein interaction (PPI) stabilizers. (**A**) Left side: crystal structure of 14-3-3ζ (white surface) in complex with peptide cystic fibrosis transmembrane conductance regulator (CFTR) R-domain pS753-pS768 (red sticks) and stabilizer fusicoccin-A (cyan sticks) [35]; right side: chemical structure of fusicoccin-A; (**B**) left side: crystal structure of 14-3-3ζ (white surface) in complex with a dephosphorylated C-RAF peptide (red sticks) and cotylenin A (cyan sticks) [43]; right side: chemical structure of cotylenin A; (**C**) right side: chemical structure of mizoribine.

The natural product fusicoccin-A (FC-A) is a metabolite produced by the fungus *Phomopsis amygdali*. It is a member of a class of diterpene glycosides bearing a 5-8-5 ring structure called fusicoccin. FC-A was the first reported stabilizer of a 14-3-3/client protein interaction [40]. FC-A was found to stabilize the 14-3-3 complex with the plasma membrane H^+^-ATPase (PMA2) with a 90-fold enhancement of the affinity between the two proteins. Structural details of the 14-3-3/PMA2/FC-A complex have been clarified via X-ray crystallography [41]. The terpene ring is buried in a hydrophobic pocket at the interface of the two proteins, while the sugar moiety is solvent exposed. Later, FC-A was found to stabilize also the binding of 14-3-3 to cystic fibrosis transmembrane conductance regulator (CFTR) (Figure 4A) and to promote its trafficking to plasma membrane [42]. Another important example is given by the stabilizing activity of FC-A toward the complex between human 14-3-3 sigma and the truncated phosphopeptide of RAF proto-oncogene serine/threonine protein kinase (C-Raf) [42]. Even if the reported systems were only approximations of the physiological complexes, as a 14-3-3 PPI stabilizer, FC-A could be a relevant tool compound for exploring the role of 14-3-3 in human diseases [30]. An example was given in a study where FC-A was used to target and stabilize the interface between ERα and 14-3-3, highlighting the potential druggability of this protein–protein interaction surface for alternative therapeutic designs in breast cancer [43].

#### 4.1.2. Cotylenin-A

Cotylenin A (CN-A) is a natural product closely related to FC-A that is produced by the fungus *Cladosporium* sp.501-7W. The crystal structure of CN-A in complex with 14-3-3 and the N-terminal binding motif of C-Raf, published in 2013 [43] (Figure 4B), provided important insights into how this small molecule stabilizes a cancer-relevant protein complex. Particularly, CN-A was found to bind the inhibitory sites of C-Raf (pSer233 and pSer259), but not the activating site pSer621, due to a sterical and electrostatic conflict between the glutamate at the +1 position from the phosphorylation site and the ring system of CN-A [43]. Furthermore, another study demonstrated how specificities for individual 14-3-3/target protein complexes might be achieved by varying the substituent pattern of the diterpene ring system [19]. As fusicoccin A and cotylenin A can play different roles in human cancers, hydroxylation of C12 might be considered as an adequate factor of structural specificity [19]. Another significant example that indicates the activity of CN-A in human cancers was given by the group of Kato, who suggested that ISIR-050 (designed as a CN-A mimic) and CN-A induce the same pharmacological response to IFNα-treated cancer cells [44].

#### 4.1.3. Mizoribine (or Bredinin)

Mizoribine (MZB) (Figure 4C) is a compound isolated from *Eupenicillium bredfedianum*. It was found to have, in vivo, inhibitory activity against the development of delayed hypersensitivity reaction to tubercle bacilli, as well as an immunosuppressive activity [45]. In vitro studies have shown that this imidazole nucleoside enhances the interaction of glucocorticoid receptors (GRs) with 14-3-3 [46].

### 4.2. Semisynthetic Fucicoccanes

**Figure 5:**
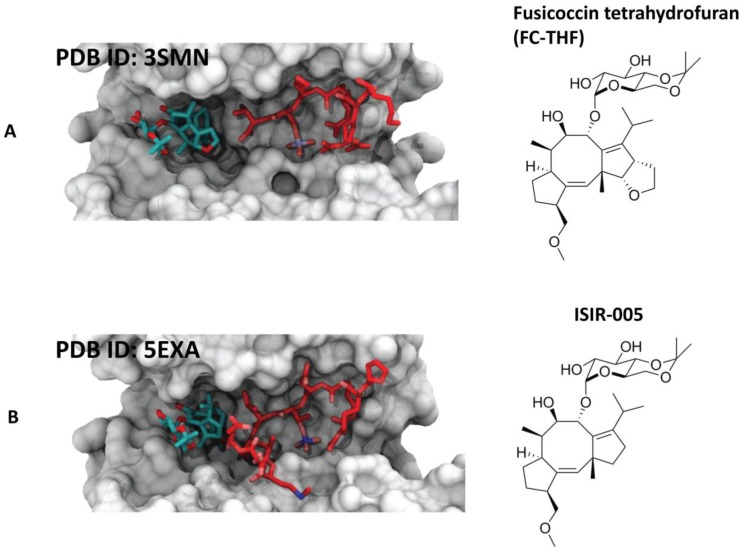
Examples of the different semisynthetic 14-3-3 PPI stabilizers. (**A**) Left side: crystal structure of 14-3-3σ (white surface) in complex with TASK-3 peptide (red sticks) and stabilizer fusicoccin A-THF (cyan sticks) [39]; right side: chemical structure of fusicoccin A-THF; (**B**) left side: crystal structure of 14-3-3ζ (white surface) in complex with Gab2 peptide (red sticks) and ISIR-005 (cyan sticks) [11]; right side: chemical structure of ISIR-005.

The 5-8-5 fused ring scaffold of fusicoccin and cotylenin is highly complex. In search for selectivity, structure-based design has instructed the semi-synthesis of potent analogues. For example, the semi-synthetic derivative FC-THF has been shown to infer a 20-fold stabilization to the complex between 14-3-3 and the potassium channel TASK-3. The derivative bearing an additional furan ring was designed as a “mode III” specific stabilizer [39] (Figure 5A). Another semi-synthetic fusicoccin-derivative (ISIR-005) has been proved to stabilize the cancer-relevant interaction of the adaptor protein 14-3-3 and Gab2. The stabilizing molecule binds to the rim of the interface of the protein complex in a pocket in the direct vicinity of the 14-3-3/Gab2pT391 interface [11] (Figure 5B).

### 4.3. Synthesis Products

#### 4.3.1. Pyrrolidone1 and Pyrazole 37

**Figure 6:**
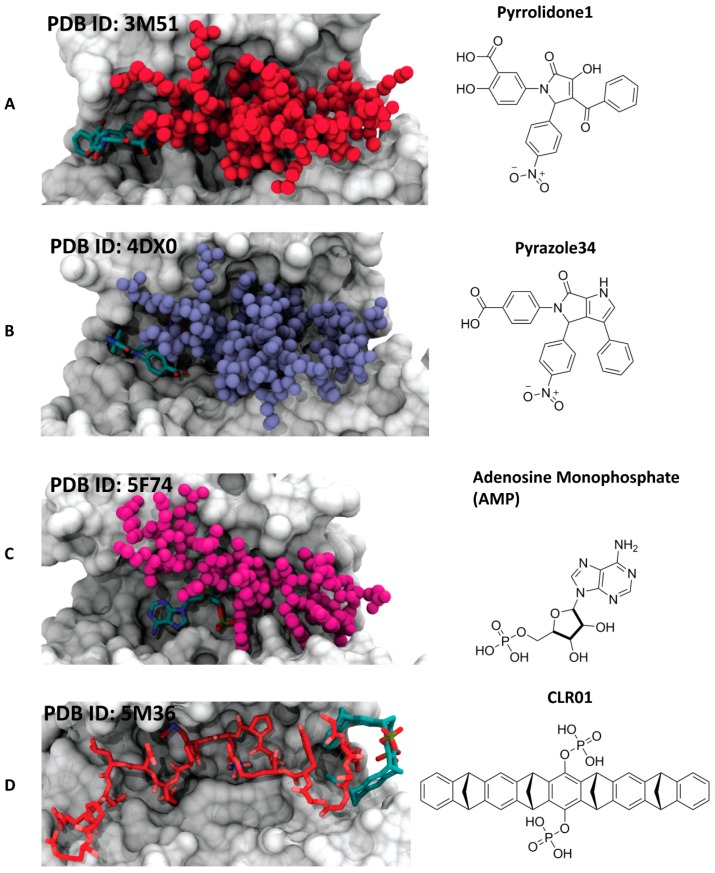
Examples of different synthetic 14-3-3 PPI stabilizers. (**A**) Left side: crystal structure of TASK-3 (white surface) in complex with plasma membrane H^+^-ATPase (PMA2) peptide (red spheres) and stabilizer pyrrolidone1 (cyan sticks) [47]; right side: chemical structure of pyrrolidone1; (**B**) left side: crystal structure of 14-3-3-like protein E (isoform of *Nicotiana tabacum*) (white surface) in complex with PMA2 peptide (ice blue spheres) and stabilizer pyrazole 34 (cyan sticks) [48]; right side: chemical structure of pyrazole 34; (**C**) left side: crystal structure of 14-3-3β (white surface) in complex with carbohydrate-response element-binding protein (ChREBP) peptide (purple spheres) and stabilizer AMP (cyan sticks) [49]; right side: chemical structure of AMP; (**D**) left side: crystal structure of 14-3-3ζ (white surface) in complex with Cdc25C peptide (red sticks) and CLR01 (cyan sticks) [51]; right side: chemical structure of CLR01.

With the aim to identify novel and chemically diverse stabilizers of 14-3-3 PPIs, in 2010, a high-throughput screening led to the identification of pyrrolidone 1. The crystal structure of the small molecule in complex with 14-3-3 and PMA2 showed how the trisubstituted pyrrolinone occupies a site that substantially overlaps with the binding pocket of FC-A [47] (Figure 6A). Starting from the pyrrolidone1/14-3-3/PMA2 crystal structure, a further optimization led to the structure of pyrazole 34 (Figure 6B). Three important modifications for the enhancement of the activity leaded the synthesis of a derivative, pyrazole37: (1) conversion of the pyrrolinone scaffold into a pyrazole, (2) introduction of a tetrazole moiety to the phenyl ring that contacts PMA2, which allows to position the stabilizer deeper into the rim of the interface, and (3) addition of a bromine to the phenyl ring that exclusively contacts the 14-3-3 protein [48].

#### 4.3.2. Adenosine Monophosphate (AMP)

Recently, AMP has been reported to stabilize the complex of 14-3-3 with the carbohydrate-response element-binding protein (ChREBP) [49]. Differently from most 14-3-3 interactions, this complex shows a phosphorylation-independent binding mechanism. The crystal structure shows how AMP occupies the same area where, in the phosphorylation-dependent 14-3-3 interactions, the phosphorylated serine or threonine is normally positioned. The small molecule establishes polar contacts with both 14-3-3 protein and the α2 helix of ChREBP, acting as a direct orthosteric PPI stabilizer (Figure 6C).

#### 4.3.3. The Molecular Tweezer CLR01

Molecular tweezers are supramolecular ligands, characterized by the alternating conjunction of benzene and norbornadiene entities. The semicircular shape of the molecule creates a cavity able to selectively accommodate long and thin side chains like lysines or arginines. In 2013, Bier et al. showed how a supramolecular ligand can modulate a 14-3-3 PPI: the lysine-specific molecular tweezer binds to a 14-3-3 adapter protein and inhibits its interaction with partner proteins, such as C-Raf and Exoenzyme S (ExoS) [50]. More recently, another study revealed that, with another 14-3-3 partner protein (Cdc25C), molecular tweezer CLR01 can stabilize the interaction. The crystal structure of the ternary complex of 14-3-3, CLR01, and the Cdc25CpS216 peptides revealed that CLR01 accommodates an arginine side chain of Cdc25C in its inner cavity and simultaneously uses its outer surface to bind into the amphipathic groove of the 14-3-3 protein (Figure 6D) [51]. Very often, protein regions involved in PPIs display a high degree of intrinsic disorder, which is reduced during the recognition process. This is also the case for the binding of the rigid 14-3-3 adapter proteins to numerous partner proteins, whose recognition motifs undergo an extensive disorder-to-order transition [51]. Cdc25C is a dual specificity phosphatase involved in the dephosphorylation in the nucleus, thus in the activation of a cyclin-dependent kinases (Cdks) and in the control of cell cycle progression and proliferation [52]. The nuclear import of Cdc25C can be negatively regulated by 14-3-3 proteins able to bind to a recognition motif enclosing the phosphorylated serine 216 (Cdc25CpS216) of the phosphatase [53]. Addition of CLR01 increased the apparent affinity of the Cdc25CpS216 peptide toward 14-3-3ζ by around 20-fold. The crystal structure of the ternary complex between 14-3-3ζ, the Cdc25CpS216 peptide, and CLR01 revealed that the molecular tweezer establishes a direct contact with the N-terminus of Cdc25CpS216 and simultaneously binds via its outer aromatic surface to the region containing residues Ser63 and Ser64 of 14-3-3ζ, the side chains of Trp59 and Tyr179, in addition to the hydrocarbon parts of the side chains of Arg60 and Glu180 [51]. This work showed the first proof of a supramolecular molecule stabilizing the binding of an intrinsically disordered recognition motif to a rigid partner protein. The molecular tweezer fills a gap in the protein–protein interface and “freezes” one of the conformational states of the intrinsically disordered Cdc25C protein partner, this way enhancing the apparent affinity of the interaction.

## 5. Inhibitors of 14-3-3 PPIs

**Figure 7:**
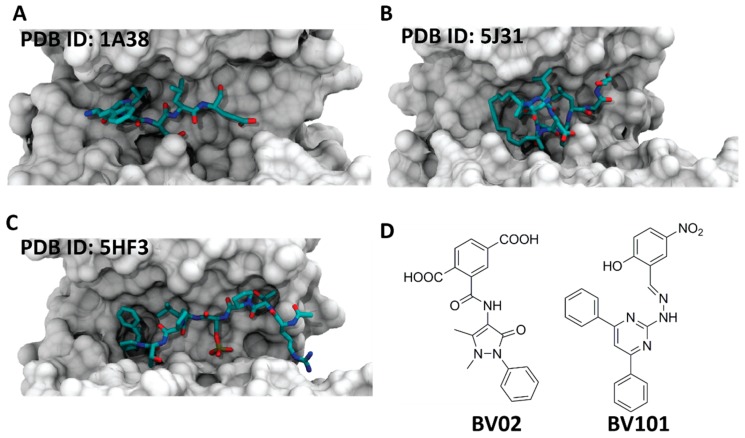
Examples of the different classes of 14-3-3 PPI inhibitors. (**A**) Crystal structure of peptide R18 (cyan sticks) in the 14-3-3ζ binding groove (white surface) (PDB ID: 1A38) [55]; (**B**) crystal structure of ExoS-derived alkyne cross-linked cyclic peptide (cyan sticks) in the 14-3-3ζ binding groove (white surface) (PDB ID: 5J31) [57]; (**C**) crystal structure of modified Tau peptide hybrid 3b (cyan sticks) in the 14-3-3σ binding groove (white surface) (PDB ID: 5HF3) [58]; (**D**) chemical structures of BV02 and BV101.

The first reported 14-3-3 inhibitor was the R18 peptide (Figure 7A), identified from a phage display [54]. The crystal structure revealed that the peptide binds the amphipathic groove of 14-3-3 and establishes polar interactions between its glutamic acid and three arginines of 14-3-3, as well as hydrophobic interactions with two leucine residues [55]. Thereafter, other peptide-based inhibitors have been developed, such as the macrocyclic peptides synthesized by the groups of Ottmann and Grossmann [56]. The inhibitors were based on the structure of the ExoS peptide (Figure 7B) and obtained with a ring-closing alkyne metathesis reaction [57]. The structure of the Tau peptide allowed obtaining another potent inhibitor (Figure 7C). The structure was designed with an extended hydrophobic area at the C-terminus that targeted the highly conserved pocket in the amphipathic groove of 14-3-3 [58]. Over the years, the implementation of computational tools has led to the development of non-peptidic 14-3-3 inhibitors. The group of Botta described the first non-phosphonate small-molecule inhibitors of 14-3-3 PPIs (BV02, BV101) (Figure 7D) by applying structure-based pharmacophore modeling, virtual screening, and molecular docking simulations [59]. Other examples are the phosphonate inhibitors identified from a virtual screening, with follow-up analysis, synthesis, and crystallization [60]. One more class of phosphate-containing molecules, called molecular tweezers, have been reported by Bier et al. The supramolecular ligand was found to bind the residue K214, which is positioned at the edge of the amphipathic group of 14-3-3 [50].

## 6. Conclusions

Modulation of 14-3-3 PPIs is a promising field in drug discovery. Understanding the role of 14-3-3 PPIs could give more insights into the cellular behavior of 14-3-3, which is essential for the development of new therapeutic strategies, such as 14-3-3 modulators. The examples reported in this review suggest how it may be possible to target certain types of disease and build up the first step towards a pharmacological intervention in human cells with aberrant cell proliferation and differentiation. In addition, all the studies reported present the stabilization of 14-3-3 PPIs as an excellent alternative to inhibitor-based standard methods, for instance, for the treatment of cancer formation and cell migration in tumors linked to malfunctioning of signal transduction processes. The stabilization of 14-3-3 PPIs is suggested to have the potential to be more beneficial compared to inhibition. In fact, the molecules identified stabilize the interactions by making contacts with both 14-3-3 and the PPI partner, therefore acting as “molecular glue”. Because of this binding mode, the stabilizers might provide a better opportunity for selectivity through the implementation of rational design approaches. Also, since the stabilizers bind to their targets in a non-competitive manner, their affinity does not need to be in the nanomolar range to trigger a strong effect in human cells. Finally, an important emerging area of investigation is the use of fragment-based drug discovery approaches. The fragments identified might be used as chemical probes to explore the surfaces of the protein partners and study the “hotspots” responsible for the binding [61]. Further efforts are needed to design more potent and selective 14-3-3 PPI modulators, which could be used to elucidate the multiple 14-3-3 functions, as well as to assess their druggability. This goal relies on the use of an extended workflow, from biophysical techniques, rational design, and X-ray crystallography to computational modeling tools.
